# Advances in ocular aging: combining deep learning, imaging, and liquid biopsy biomarkers

**DOI:** 10.3389/fmed.2025.1591936

**Published:** 2025-07-23

**Authors:** Dengren Zhang, Naiyang Li, Fan Li

**Affiliations:** ^1^Eye Center, Zhongshan City People’s Hospital, Zhongshan, China; ^2^Shenzhen University Medical School, Shenzhen, China; ^3^Key Laboratory of Regenerative Medicine, Ministry of Education, Jinan University, Guangzhou, China; ^4^The First Clinical Medical College, Guangdong Medical University, Zhanjiang, Guangdong, China

**Keywords:** ocular aging, deep learning, liquid biopsy, imaging, age-related eye diseases

## Abstract

Ageing is a significant risk factor for a wide range of human diseases. Yet, its direct relationship with ocular ageing as a marker for overall age-related diseases and mortality still needs to be explored. Non-invasive and minimally invasive methods, including biomarkers detected through ocular imaging or liquid biopsies from the aqueous humour or vitreous body, provide a promising avenue for assessing ocular ageing. These approaches are particularly valuable given the eye’s limited regenerative capacity, where tissue damage can result in irreversible harm. In recent years, artificial intelligence (AI), particularly deep learning, has revolutionized medical research, offering novel perspectives on the ageing process. This review highlights how integrating deep learning with advanced imaging and liquid biopsy biomarkers has become a transformative approach to understanding ocular ageing and its implications for systemic health.

## 1 Introduction

Ocular aging constitutes a multifaceted and intricate process, entailing a successive cascade of structural and functional metamorphoses. Ocular aging is not the same as the aging or biological age of other organs. In the anterior segment, the cornea undergoes alterations such as a diminution in endothelial cell density and a perturbation in collagen fibril organization, which potentially culminate in impairments to corneal transparency and refractive power. Scholarly investigations have divulged that, after 40, the corneal endothelial cell density wanes slowly (1). Likewise, succumbing to the inexorable passage of time, the lens accrues crystalline and other proteins, precipitating cataract formation and a consequent loss of accommodative faculty. By the age of 80, over half of the populace will have manifested cataracts to some degree (2). In the posterior segment, the retina and choroid encounter their own set of tribulations. The retinal pigment epithelium progressively forfeits its efficacy in phagocytosing photoreceptor outer segments, while the choroid attenuates and its vascular supply becomes less efficient, both of which can undermine retinal function and visual acuity (3).

The advent of deep-learning imaging has instigated a revolutionary transformation within the realm of ophthalmology. By capitalizing on the potency of convolutional neural networks and other advanced machine learning architectures, extracting exquisitely intricate details from ocular images has become feasible. High-resolution optical coherence tomography (OCT) images can now be dissected to unearth minuscule changes in retinal layer thicknesses, microstructural irregularities, and even incipient signs of macular degeneration. When applied to OCT images, DL algorithms could detect early signs of macular degeneration with an accuracy surpassing 80%, which has been convincingly demonstrated (4). When amalgamated with deep learning algorithms, fundus photography can identify fine vascular alterations and patterns that correlate with ocular aging and disease progression. This technological innovation not only augments the sensitivity and specificity of diagnosis but also facilitates the earlier detection of latent problems, potentially paving the way for more opportune interventions.

Concomitant with this imaging revolution, liquid biopsy biomarkers have emerged as a highly promising avenue in ocular aging research. Bodily fluids like blood and aqueous humor harbor a cornucopia of biomolecules that can serve as proxies for the physiological state of the eye. Proteins, whether secreted or shed from cells, can also mirror changes in ocular tissue metabolism and inflammation. In a recent proteomics study, researchers ascertained that proteins in the aqueous humor could prognosticate ocular aging, and patients afflicted with certain eye diseases exhibited proteins that signified a markedly higher age compared to healthy individuals (5). By dissecting these biomarkers, we can plumb the depths of the molecular underpinnings of ocular aging and potentially identify novel therapeutic targets.

The integration of deep learning imaging and liquid biopsy biomarkers epitomizes a paradigm shift in ocular aging research. On one hand, deep learning imaging can orchestrate the sampling of body fluids by precisely pinpointing areas of concern within the eye. When a deep learning model discerns a suspicious region in the retina, it can prompt the collection of aqueous humor from the corresponding area to search for relevant biomarkers. On the other hand, biomarker data can be fed back into the deep learning models to augment their predictive capabilities. If a particular biomarker is associated with a certain aging or disease phenotype, the model can be trained to recognize the imaging features that accompany it. This symbiotic relationship between the two approaches has the potential to unlock novel diagnosis strategies, which is precisely the motivation underpinning this comprehensive exploration of the advances in this integrated field.

## 2 Application of deep learning in anterior segment aging

### 2.1 Deep learning in ocular anterior segment aging

Many anterior segment ocular diseases are closely associated with the aging process. Deep learning technology has been harnessed to engineer automated diagnostic systems, such as feature extraction and classification networks predicated on deep learning architectures, for the identification and management of anterior segment diseases. An age prediction model was erected based on a copious quantity of correlated features gauged by an anterior segment analyzer. Subsequently, three DL modalities (neural network, Lasso regression, and extreme gradient boosting) were employed to unearth the alterations in the estimated actual age contingent upon anterior segment morphology (6).

**FIGURE 1:**
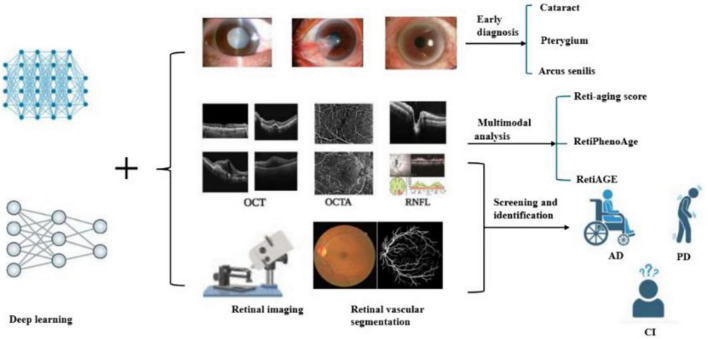
Retinal imaging techniques such as OCT (optical coherence tomography), OCTA (optical coherence tomography angiography), and RNFL (retinal nerve fiber layer) are combined with deep learning algorithms to analyze eye conditions. The process involves identifying cataracts, pterygium, and arcus senilis, and calculating a Reti-aging score using multimodal analysis. Additionally, it aids in screening and identifying conditions like age-related macular degeneration (AMD), Parkinson’s disease (PD), and cognitive impairment (CI).

Deep learning has become a leading sentinel in diagnosing diseases. Deep learning models have been enlisted to automatically assimilate and appraise the severity of cataract. The automatic grading of nuclear cataracts has been actualized by meticulously scrutinizing anterior segment images. A hybrid global-local representation convolutional neural network (CNN) model based on DL principles has been devised for the automated categorization of cataracts (7) (Figure 1). In addition, deep learning also has strong performance in identifying degenerative keratopathy such as corneal rings, and the proposed model achieves 100% accuracy in identifying ocular images into normal and AS categories by using AlexNet as a pre-trained network for transfer learning (8).

AI has increasingly been used in the assessment of the anterior segment, precision in anterior chamber and iris tissue measurements (9) (Figure 1). Presbyopia, a typical vision impairment caused by aging, has an insidious onset but a widespread impact. Deep learning models integrate anterior segment biometric data, including dynamic parameters such as lens elastic modulus and ciliary muscle contraction amplitude (10) (Figure 1). Pterygium is an ocular surface disease induced by the synergistic effect of long-term ultraviolet radiation, wind and sand stimulation and aging, which affects the microenvironment of limbal stem cells, which may lead to damage to limbal stem cells (11). In recent years, the three types of intelligent lightweight auxiliary pterygium diagnostic models using the MobileNet model combined with anterior segment images have greatly improved the sensitivity and specificity of disease diagnosis (12).

### 2.2 Ocular surface biomarkers associated with aging

A novel category of non-invasive visual aging biomarkers, namely PhotoAgeClock, which solely employs anonymous images of the eye corners, has ascertained that high-resolution imagery proves to be highly advantageous when estimating age from facial images. Notably, wrinkles and skin pigmentation have emerged as dependable non-invasive visual biomarkers of aging, thereby endowing them with the potential resource for discerning the condition and health status of the human body. When PhotoAgeClock was harnessed for age prediction on an anonymous image dataset, it was unveiled that, compared to other regions within the surveyed images, the skin encircling the eyes as the area exhibiting the highest correlation with age (13).

Another important point is the tear, which contains many proteins, cytokines, and other substances that participate in the regulation of the function and metabolism of various cells in the eye, and then reflect the pathophysiological state of the individual, which has a certain role in predicting aging (14). In tear samples, the levels of some inflammatory factors, such as MCP-1 and IL-6, were positively correlated with the incidence of age-related diseases such as cataracts (15). Tear-related proteomics showed a high correlation with age, with seventeen tear proteins that showed a significant correlation with age, including cytoplasmic actin (ACTB and ACTG1), albumin (ALB), and annexin A1 (ANXA1), which were positively associated. Additionally, proteins such as ubiquitin-like modification-activating enzyme 1 (UBA1) and Golgi membrane protein 1 (GOLM1) were identified. Furthermore, some transcriptional regulators, including the activation of NF-κB, are observed in the tears and aging ocular surface (16).

In addition, the expression levels of p16INK4a and p21CIP1 in senescent corneal cells increased with age. These factors are often associated with cell cycle arrest and are important markers of cellular senescence. Senescent corneal cells secrete a variety of pro-inflammatory factors such as IL-6, IL-1β, MMP-3, MMP-9, and TNF-α. Increased secretion of these factors is associated with an ocular inflammatory response, which may lead to inflammation and dysfunction of the corneal surface. The study found that aging corneal cells secreted GDF15 increased, while LTBP3 levels decreased. This alteration may lead to the activation of TGF-β, which in turn triggers a cascade of cellular senescence-related responses, such as the activation of NF-κB and secondary cellular senescence, exacerbating the inflammatory response. Senescent corneal cells exhibit features of increased keratinization and decreased neurophenotyping. This may indicate that keratinization and neural phenotype-associated proteins are involved in corneal cell differentiation and functional alterations, affecting the normal physiological function of the cornea (17).

## 3 Application of deep learning to aging retinal images in the fundus

### 3.1 Identification and diagnosis related to aging diseases

Deep learning technology has been extensively deployed to automatically identify and classify retinal aging-related ailments, such as diabetic retinopathy (DR) and age-related macular degeneration (AMD). These advanced technologies possess the prowess to autonomously categorize pathologies and diseases manifesting in retinal images, thereby remarkably augmenting the efficiency of early diagnosis and impeding the progression of diseases. The application of transfer learning in the automatic classification of retinal images has also been the subject of extensive research. By fine-tuning pre-trained models on large-scale image classification datasets like VGG, InceptionV3, MobileNetV2, ShuffleNetV2, and GAN, OCT retinal images can be efficaciously categorized, enabling the automatic classification and diagnosis of diabetic macular edema, age-related macular degeneration (18, 19).

Yow et al. suggested a new methodology aimed at differentiating the retinal vascular and neuronal elements in the retinal nerve fiber layer (RNFL) for thickness assessment. This is achieved by effectively integrating structural and vascular data obtained from OCT and OCT angiography (OCTA) images (Figure 1). The method utilizes the segmented RNFL from cross-sectional OCT imaging alongside blood flow information from enface OCTA imaging to accurately determine the positions of major vessels and microvasculature in both lateral and axial dimensions. Furthermore, an analysis of RNFL thickness in relation to the vessel proportion revealed a notable relationship between thickness and age (20).

As we age, the thickness of the RNFL usually becomes progressively thinner. This change may be related to the natural aging and apoptosis of retinal ganglion cells, resulting in a decrease in the number of nerve fibers, which in turn thins the RNFL (Figure 1). Thinning of the RNFL may affect the function of the optic nerve, leading to problems such as vision loss and visual field defects, which is an important factor in visual dysfunction during aging. Age is inversely correlated with vascular density in the macula. As individuals age, there is a reduction in the vascular density of the deep capillary plexus (DCP) and the superficial capillary plexus (SCP) within the macula (21). Moreover, it was generally observed that when all detected vessels were excluded, the average thickness profiles along the micropapillary RNFL in healthy subjects exhibited a lower absolute variability. This innovative approach potentially holds the key to enhancing the precision of diagnostics and the enhancement of identifying and measuring neuronal and vascular degeneration in individuals suffering from eye-related conditions like glaucoma and diabetic retinopathy (20).

In studies where convolutional neural networks (CNN) were amalgamated with OCTA, it was established that retinal vascular density and fractal dimension (FD) were correlated with event mortality. Particularly in patients with type 2 diabetes or hypertension, low FD and vascular density were conspicuously related with a higher likelihood of prevalent and incident cardiometabolic phenotypes (22). Furthermore, a reduction in the density of retinal blood vessels was notably linked to both overall mortality and premature death. A research project utilizing the UK Biobank database discovered that a reduction in retinal vascular density was notably linked to both overall mortality and early death, even after considering demographic, health, and lifestyle variables (23). There exists a significant association between the retinal vasculature system and both systemic and ocular diseases, encompassing aging-related diseases such as myopia, age-related macular degeneration, retinal detachment, glaucoma, and cataracts (Figure 1).

### 3.2 Application of multimodal analysis in fundus aging prediction

Multimodal data fusion is an important development direction for the diagnosis of retinal aging. By combining multiple imaging modalities such as OCT and fundus images, deep learning models can more comprehensively assess the aging status of the retina and improve the accuracy of diagnosis. Multimodal imaging technology can achieve the fusion of OCT and fundus images, and OCT can provide high-distinguishability cross-sectional images of the retina, revealing subtle structural changes in the retinal layer. Fundus images, on the other hand, can visualize the overall morphology and vascular distribution of the retina. Combining these two imaging modalities, the deep learning model can more comprehensively identify the features of retinal aging.

Multimodal analysis combined with multiple imaging techniques (fundus images, OCT, OCTA, etc.) can provide richer information for predicting biological age and assessing the risk of aging-related diseases. For example, one study developed a Transformer-based multimodal architecture to estimate biological age by integrating images of the face, fundus, and tongue. The results suggest that the fusion of multiple image modalities can achieve more accurate biological age prediction and the variance between biological age and chronological age (AgeDiff) may serve as a promising biomarker for the stratification of chronic disease risk (24). In one study, VisionFM is a multimodal, multitasking, vision-based model capable of handling multiple aging-related ophthalmic diseases and imaging modalities. The model excels in a variety of fundus disease diagnoses, including DR and AMD, and can predict glaucoma progression and the presence of intracranial tumors from fundus images (24). In addition, retinal vein occlusion is a retinal vascular disease associated with vascular degeneration and dysfunction. A research effort created a deep learning model that utilizes multiple modalities to predict branching retinal vein occlusion (BRVO) by integrating fundus images with features of retinal vasculature. The model significantly improved prediction accuracy by analyzing the retinal vascular structure (25) (Figure 1).

Deep learning models can also be used to predict vascular aging from retinal fundus images related to retinal aging closely. Prediction of vascular aging involves utilizing deep learning models to assess vascular aging based on retinal fundus images. Combined with clinical parameters for training, the model shows high potential in identifying new hypertension and carotid plaque. In addition to imaging techniques, some studies have combined transcriptomics and proteomics to analyze gene and protein expression changes in the retina during aging. Research indicates that proteins specific to photoreceptors undergo changes prior to a decline in function, suggesting a possible biomarker for the early detection of retinal aging (26).

### 3.3 Multi-disease screening and identification by integrating deep learning with ocular imaging

The analysis of the retinal vasculature assumes a pivotal role in the diagnosis of numerous ocular and systemic diseases, and recent research has highlighted its potential significance in the context of neurodegenerative diseases. Retinal microvasculature shares similar anatomy and physiology with cerebral vasculature, making it a valuable non-invasive window for studying neurovascular changes associated with neurodegenerative conditions. Against this backdrop, changes in retinal thickness can be used to accurately predict some neurodegenerative diseases in combination with the deposition of fundus-related pathological proteins (27). In addition, accurate detection of vascular features has been modeled as multi-instance heatmap regression in fundus images. Deep learning models, such as NFN + neural networks, can extract vascular features from raw fundus images to improve accurate identification and prediction of associated cognitive dysfunction (28).

#### 3.3.1 Application for Alzheimer’s disease (AD)

Deep learning models have demonstrated a remarkable capacity not only to identify retinal diseases with precision but also to concurrently screen and discern multiple systemic neurodegenerative conditions. This dual functionality is of paramount importance as it empowers physicians to make preliminary and astute judgments, especially in scenarios where a single patient may present with a complex array of comorbidities. Relevant researchers have indicated that the characteristic pathological signs of Alzheimer’s Disease (including Aβ and pTau accumulation) can emerge in the retina of affected individuals, which provides unique insights into the preclinical progression of the disease, and retinal amyloid/tau pathological imaging is the frontier direction of AD biomarker research. Some studies have analyzed structural changes in the retina to look for biomarkers associated with AD through OCT and OCTA techniques (29). In recent years, studies have found that changes in retinal microvessels are closely related to the early pathology of AD. Microvascular alterations within the retinal deep vascular complex (DVC) are recognized as significant indicators of cognitive decline. Recent research introduces an innovative deep learning model known as Eye-AD, designed specifically to identify the early stages of Alzheimer’s disease (EOAD) and mild cognitive impairment (MCI) by utilizing OCTA images. This advancement represents a promising approach to diagnosing cognitive deficits at their onset, potentially improving early intervention strategies. The model uses a multilayer graph neural network (GNN) to analyze the relationship between different layers of the retina, thereby improving detection performance. Eye-AD performed well in distinguishing AD patients from healthy controls, with an AUC value of 0.93. Another study developed a deep learning-based classification system capable of distinguishing AD patients from healthy individuals by analyzing changes in the retinal vascular network (30). Retinal imaging techniques, such as OCTA, have been shown to detect microvascular changes associated with AD. For example, increased tortuosity of retinal veins is associated with the presence of β-amyloid plaques in the brain. In addition, thinning of the RNFL and reduction of retinal vascular density are also considered potential biomarkers for AD (31) (Figure 1).

The hallmark pathological manifestations of AD, namely Aβ and pTau deposition, may potentially transpire in the retina of AD patients (32), thereby proffering unique insights into the preclinical progression of the disease. Herein, we undertake a comprehensive review of current research pertaining to imaging amyloid/tau pathology in the retina and explore the implications for the development of retinal biomarkers of AD (33). In addition, retinal thickness (TRT) in animal models of AD has been shown to be associated with the prediction of age and aging. A research endeavor employing OCT to scrutinize TRT of wild-type (WT) and 3 × Tg-AD mice (Alzheimer’s disease models) unearthed that the TRT of both groups diminished with age. Remarkably, the retina of WT mice was conspicuously thicker than that of 3 × Tg-AD mice at all ages, barring the 16-month mark. Two convolutional neural network models were trained to prognosticate the age of mice from OCT B-scans, signifying that the age of mice could be reasonably and accurately predicted. Moreover, the retinal aging patterns of WT and 3 × Tg-AD mice diverged, with projections indicating that the age of WT retinas would exceed that of 3 × Tg-AD retinas after the fourth month (34).

#### 3.3.2 Application for Parkinson’s disease (PD)

A network predicated on frequency domain learning has been devised for automatic Parkinson’s disease (PD) screening. By amalgamating prior clinical knowledge, it automatically zeroes in on specific retinal structures (RNFL, GCIP, OPL, ONL), thus circumventing the challenge of PD being arduously identifiable due to the subtle alterations in retinal structures. A module was meticulously designed using Discrete Wavelet Transform (DWT), integrating low-frequency and high-frequency domain information and capitalizing on the unique traits of each frequency band to accurately screen for PD (35). Recent clinical investigations have hinted at retinal layer atrophy and decreased microvascular density in individuals with Parkinson’s disease (PD), predominantly detected via OCT and OCTA image. These modalities have been utilized to assess classification accuracy throughout various stages of PD progression, specifically incident (encompassing pre-symptomatic or prodromal) and prevalent PD. The Automorphy deep learning segmentation algorithm was employed to delineate key retinal components, including arteries, veins, optic cup, optic disk, and a manually annotated fovea. Notably, changes in the size, thickness, and other attributes of the optic cup, optic disk, and fovea have been identified as indicators of PD progression (36) (Figure 1).

#### 3.3.3 Application for cognitive impairment (CI)

The NFN + deep learning model has been employed to analyze retinal vessel characteristics for the recognition of CI. Ocular examinations can serve as a non-invasive screening implement to explore pathological changes in brain microcirculation and relevant cognitive impairment. Rui Li et al. revealed a tendency to have a positive correlation between cognitive function and retinal vascular fractal dimension (FD). In contrast, a negative correlation exists between cognitive function and global vein width. The study harnessed random forest to prognosticate the development of cognitive decline. The order of importance of predictors in the model was as follows: age (0.193), Body Mass Index (BMI) (0.154), global vein width (0.106), retinal vessel FD (0.099), and Central Retinal Artery Equivalent (CRAE) (0.098). The area under the curve (AUC) values predicted by the model were 0.799. The NFN + model, which can extract vascular features from fundus images, exhibits a high degree of recognition and predictive prowess for cognitive function and can be utilized as a screening modality for CI (28).

These results also suggest that changes in the complexity of fundus blood vessels—such as fractal dimension (FD) and vein width—could be useful as potential biomarkers for cognitive dysfunction. Since the retina and brain share similar vascular development and characteristics, changes in retinal blood vessels may reflect the condition of the brain’s microcirculation. This makes fundus vascular measurements a promising, non-invasive method for early screening of cognitive issues. However, it’s important to recognize that fundus aging doesn’t always match systemic or biological aging. The condition of blood vessels in the retina can be affected by various factors, including genetics, environment, lifestyle, and diseases like hypertension and diabetes. Therefore, retinal aging may provide a unique perspective on both local and overall aging processes.

These findings highlight the potential of the retina not only as a window into brain health but also as a valuable tool for studying aging (28) (Figure 1). Deep learning models can help distinguish between normal retinal aging and early signs of disease by analyzing changes in thickness, vessel structure, and other features. This is important because aging and disease can cause similar changes in the retina. With AI, it’s possible to detect both patterns—those related to natural aging and those linked to diseases like Alzheimer’s—at the same time. This approach helps improve early diagnosis and also gives us a better understanding of how aging affects the eye and overall health.

## 4 Retinal imaging biomarkers associated with deep learning in aging diseases

### 4.1 RetiPhenoAge

RetiPhenoAge manifests as a cutting-edge, deep-learning-empowered biological aging marker. It astutely amalgamates retinal images with PhenoAge, a comprehensive biomarker emblematic of phenotypic age, for the purpose of prognosticating morbidity and mortality rates with an unprecedented level of precision. The cardinal aim of this seminal investigation is to anticipate the vicissitudes associated with kidney function, immune function, liver function, inflammation, energy metabolism, and chronological age. This is accomplished by meticulously discerning retinal patterns and nuanced characteristics that are inextricably linked to the fluctuations in blood biomarkers.

The study has unearthed a remarkable revelation: for each incremental augmentation of 1 year in the retinal age gap, there exists an independent and statistically significant correlation with a 10% elevation in the risk of incident PD. The retinal age gap has incontrovertibly emerged as a reliable and valid biomarker of aging, endowing it with the capacity to prognosticate mortality risk and presenting itself as a highly promising candidate for the early detection of PD (37). Intriguingly, a conspicuous positive mean differential has been observed in the retinal age gap between diabetic patients afflicted with DR and those devoid of the condition. Moreover, this disparity progressively amplifies in tandem with the exacerbation of DR severity. These outcomes potentially intimate an underlying nexus between the pathological evolution of the disease and the premature senescence of the retina (38). The latest research findings have accentuated a correlation between the retinal age gap, computed via a state-of-the-art deep learning model, and metabolic syndrome (MetS) as well as inflammation. Specifically, the more pronounced the retinal age gap, the greater the susceptibility of individuals to developing MetS and its constituent elements (abdominal obesity, hypertension, hyperglycemia), accompanied by an upsurge in inflammatory markers. In comparison to those individuals exhibiting the narrowest retinal age gap, those with a more substantial gap face an augmented risk ranging from 10 to 27% for abdominal obesity and hypertension, 14%–19% for hypertension in isolation, and a staggering 25%–104% for hyperglycemia (39). Moreover, it can be efficaciously harnessed to dissect the relationship with the incidence of chronic kidney disease (CKD) and cardiovascular disease (CVD) (40, 41).

### 4.2 RetiAGE and reti-aging

RetiAGE, as an alternative yet superlatively efficacious biomarker, has demonstrated exemplary performance in predicting the mortality of CVD and related pathologies. By homing in on the macula, optic disk, and retinal vasculature, and synergistically integrating fundus images, the predictive potency of the risk model is exponentially enhanced with the incorporation of DL-predicted RetiAGE scores (42). The deep learning retinal vascular aging score, denominated as the Reti-aging score and proffered by the Reti-aging scoring model, has emerged as a novel and revolutionary methodology for predicting vascular aging through the painstaking analysis of retinal fundus images. It furnishes a novel metric that augments the prognostic capabilities for the incidence of cardiovascular diseases (32) (Figure 1).

### 4.3 Extraocular biomarkers combined with retinal imaging techniques

Furthermore, retinal imaging can be synergistically conjoined with other biomarkers such as inflammatory markers (e.g., IL-6, TNF-α) and metabolic markers (e.g., HbA1c, lipid profiles) to conduct a holistic and comprehensive appraisal of the body’s health status. Utilizing retinal images as input, a deep learning (DL) model can calculate an individual’s cardiac BioAge. This approach enables the DL cardiac BioAge model to effectively categorize individuals according to conventional cardiovascular disease risk biomarkers (43).

## 5 Ocular aging at the molecular level

### 5.1 Corneal aging under sunlight exposure

Quantitative scrutiny of the ocular structures pertaining to mtDNAT414G divulged an age-correlated accretion of mtDNAT414G within the corneal stroma. The mtDNAT414G mutation, which stems from cumulative solar exposure, holds the potential to serve as a reliable biomarker of solar exposure. As the frequency of mtDNAT414G mutations mounts, the ocular aging process unfolds, rendering it feasible to amalgamate these disparate levels of mtDNA mutations, thereby facilitating a precise measurement of the total solar radiation exposure an individual has experienced over their lifetime (44).

### 5.2 Molecular mechanisms of age-related ocular diseases

Several age-related ocular conditions have been identified as having strong associations with molecular and structural changes observed during ocular aging. Diseases that manifest associations in this context are AMD and cataracts. AMD represents a localized malfunction within the macular photoreceptors/retinal pigment epithelium/Bruch’s membrane/choroid-capillary complex, culminating in photoreceptor attrition and, ultimately, central vision impairment. It stands as an almost quintessential hallmark of aging-induced ocular degeneration (45). The etiological mechanism underpinning cataract genesis is multifaceted, encompassing oxidative stress, protein denaturation, DNA damage and repair inefficacy, as well as perturbations in lens epithelial cells. Oxidative stress occupies a central position in lens senescence and cataract formation, given that lens epithelial cells orchestrate key metabolic activities and maintain intimate connections with lens fibroblasts via interstitial junctions. Consequently, they exhibit heightened susceptibility to the metabolic perils besetting lens epithelial cells, especially those of oxidative origin (46, 47). Age-related cataracts (ARCs) have been linked to deficiencies in DNA repair mechanisms within lens epithelial cells (LECs). Reactive oxygen species (ROS)-induced DNA damage is postulated to be a pivotal determinant in ARC development (48).

In a parallel vein, oxidative stress is also posited as a cardinal factor in the pathogenesis of AMD (45). Dysregulation of the complement system emerges as a significant impetus driving AMD pathogenesis, as immune-mediated damage, consequent to excessive complement activation, expedites the progression of the disease (49). Cellular senescence represents an important predisposing factor for AMD, since cells transition into a permanent cell cycle arrest state following a finite number of divisions. With the inexorable march of age, the population of senescent cells swells and exhibits a robust correlation with a plethora of age-related chronic maladies (50).

Ocular aging at the molecular level is a complex process involving dynamic changes in various cellular and molecular pathways. Recent advancements in liquid biopsy techniques, combined with proteomics, have provided unprecedented insights into the molecular mechanisms underlying eye aging. By analyzing biomarkers in ocular fluids such as aqueous humor or vitreous humor, researchers can now identify specific proteins and cellular signatures associated with normal aging and age-related ocular diseases. This integration of liquid biopsy biomarkers with proteomics not only enhances our understanding of the molecular trajectory of ocular aging but also offers potential for early detection, diagnosis, and personalized treatment strategies for age-related ocular conditions (51).

The relevant study has found that telomere shortening and declining NAD + levels are key molecular mechanisms of aging as we age. Telomere dysfunction activates the DNA damage response, leading to cell cycle arrest, apoptosis, and aging, which in turn leads to chronic inflammation and age-related diseases. In addition, the decrease in NAD + levels can affect cellular energy metabolism and accelerate the aging process. In the field of ophthalmology, the combination of liquid biopsy and proteomics provides new tools for early detection and intervention of age-related eye diseases. By detecting biomarkers in intraocular fluid, apoptosis of retinal ganglion cells, changes in retinal vascular density, and decreased optic nerve function can be identified earlier. Changes at these molecular levels may occur before clinical symptoms appear, so early intervention is essential to slow disease progression (52).

## 6 Liquid biopsy biomarkers combined with proteomics in eye aging

**FIGURE 2:**
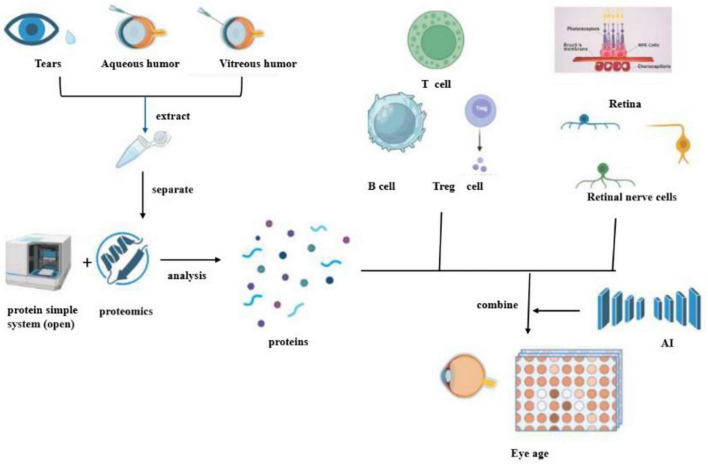
This diagram outlines a process for analyzing proteins in eye fluids to determine eye age. It begins with the extraction of proteins from tears, aqueous humor, and vitreous humor. These proteins are then separated and analyzed using proteomics, which involves a simple protein system (open). The identified proteins are associated with various immune cells, such as T cells, B cells, and Treg cells, as well as retinal and retinal nerve cells. The data is combined with artificial intelligence (AI) to assess eye age, potentially offering insights into ocular health and aging.

An in-depth cell-derived analysis, which entailed the integration of proteomics from liquid biopsy and single-cell transcriptomic analysis of diverse ocular cell types, was conducted on 5,953 proteins detected in the aqueous humor. This comprehensive investigation unveiled that liquid biopsy, when synergistically combined with proteomics and deep learning, holds the potential to vigilantly monitor and accurately diagnose a spectrum of complex disorders, including retinitis pigmentosa, diabetic retinopathy, Alzheimer’s disease, and Parkinson’s disease. It thereby proffers novel and promising targets for the early detection of age-related retinopathy and degenerative neurological ailments. Moreover, leveraging advanced AI models, the overall molecular age as well as the cellular molecular age of the eye was predicted. Notably, endothelial cells and the retinal pigment epithelium/choroid complex in the retina emerged as the principal contributors to AI vascular markers. Meanwhile, B cells, vitreous cells, and T cells were identified as the driving forces behind AI Immunosenescence markers, while bipolar cells, rod cells, and ganglion cells played a pivotal role in powering the AI retinal senescence models (51) (Figure 2).

Through the application of liquid biopsy technology, protein molecules associated with aging have been discerned in tears, aqueous humor, and vitreous humor. Subsequently, an AI protein circadian clock has been ingeniously developed using TEMPO technology. Intriguingly, these AI clocks have demonstrated the capacity to predict the age of corresponding cell types, such as blood vessels, retinal cells, or immune cells. When the AI circadian clock was applied to diseased eyes, it was observed that ailments like diabetic retinopathy and uveitis could precipitate accelerated aging of specific cell types. Proteomics has proven to possess high diagnostic value, especially in the detection of specific proteins during the analysis of age-related retinal inflammatory diseases. Previous studies have meticulously examined liquid biopsies from the fovea, macula, and beneath the peripheral retina within the proteome of the human choroid-retinal pigment epithelium (RPE). The findings of these studies revealed that, on average, 4403 unique proteins were identified in each fovea, macula, and peripheral choroidal RPE tissue. Among these, the 671 differentially expressed proteins encompassed risk factors for retinal diseases linked to oxidative stress, inflammation, and the complement cascade. Significantly, the complement cascade represents a potential therapeutic target for AMD (53).

Furthermore, related research has indicated that immune cells predominantly comprise T cells present in the vitreous cavity, as ascertained through vitreous fluid biopsy. Notably, neutrophils were conspicuously absent, while a comprehensive repertoire of adaptive T cells, including CD4+, CD8+, regulatory T cells (Treg), and innate immune system effector molecules (i.e., natural killer T cells), were detected in the vitreous humor. These findings suggest CD4+ and CD8+ activation of memory T cells and the occurrence of vitreous-specific ligand receptor interactions. By harnessing the potential of vitreous humor biopsy, the revelation of the immune system’s involvement in regulatory mechanisms has furnished invaluable insights into the etiological underpinnings and potential treatments of vitreoretinal-related aging diseases in humans (54). At the cellular and tissue levels of eye diseases, remarkable strides have been made in elucidating age-related changes. Mounting evidence substantiates the fact that both innate and recruited immune cells play a crucial role in modulating innate immunity in eye diseases, a phenomenon that is increasingly being recognized as a pivotal factor in the pathogenesis of both infectious and non-infectious eye diseases (55) (Figure 2).

Eye aging in certain animal models, such as fruit flies, exhibits some parallels with human eye aging. In a particular study focused on fruit flies, researchers delved into proteomic alterations in aging eyes. By employing proteomics techniques, they discovered that the overall changes in protein abundance in the fruit fly eyes mirrored the collective contributions of multiple cell types, potentially obscuring the more subtle changes in individual cell types, such as photoreceptors. Additionally, the discrepancies between the senescent transcriptome and proteome in the eye might signify changes in protein synthesis or post-translational abundance, rather than being solely attributable to transcript abundance (56).

A comparative analysis of diagnostic methods for ocular aging was conducted, focusing on deep learning-based imaging, traditional imaging techniques, and liquid biopsy biomarkers. These approaches differ in terms of accuracy, cost, invasiveness, and potential for early detection. A detailed comparison is presented in Table 1.

**TABLE 1:** Comparison of diagnostic methods for ocular aging.

Evaluate dimensions	Deep learning Imaging	Traditional imaging	Liquid biopsy biomarkers
Technical principle	CNN/GNN and other algorithms to multi-modal images (OCT, fundus photography, OCTA)	Manual interpretation of single-modal images (e.g., slit lamp, basic OCT)	Detect proteins, metabolites, inflammatory factors, etc. in tears/aqueous humor (15)
Accuracy	High (AUC 0.85–0.95, as detected by the Eye-AD model) (30)	Moderate (physician experience-dependent, AUC 0.65–0.80)	High (specific proteins such as IL-6 are associated with aging > 0.9) (15)
Early detection capabilities	Identification of subclinical microstructural changes (e.g., thinning of RNFL)	Only visible lesions can be detected	Early changes at the molecular level may be detected
Cost	Medium (requires high-performance computing equipment, but can be used at scale)	Low (device widespread)	High (high cost of mass spectrometry/sequencing, invasive sampling) (51)
Viability	Datasets and algorithm training need to be labeled	Routine clinical application	Depending on laboratory conditions, limited sampling (e.g., risk of aqueous humor puncture) (5)
Advantage	Multimodal data fusion; Automate screening of large populations	Simple operation; Immediate diagnosis	Revealing molecular mechanisms; Discovery of potential therapeutic targets
Limitations	The black box model has poor interpretability; Risk of data bias	Subjectivity; Limited resolution	The dynamics of biomarkers are highly variable; Standardization is difficult
Typical applications	Retinal Age Prediction (RetiPhenoAge) (37–39); neurodegenerative diseases screening	Cataract grading; Glaucoma optic disk assessment	mtDNA mutation detection for corneal senescence (44); Inflammatory factor monitoring

## 7 Discussion

Ocular aging can include ocular surface senescence and fundus senescence related to deep learning technology, in which deep learning technology has been increasingly integrated into the evaluation and diagnosis of ocular surface aging-related diseases, but in liquid biopsy, the proteome of ocular surface biomarkers such as tears and relevant inflammatory factors are not particularly clear related to aging. Aqueous humor sampling is invasive and risks infection, restricting longitudinal studies. No universal consensus exists for biomarker quantification, complicating inter-study comparisons.

Deep learning can be used for preoperative planning and intraoperative navigation. For example, models based on deep learning can analyze the anatomical structures of the anterior segment of the eye and provide precise recommendations for the location and depth of incisions in cataract surgery. Deep learning can also monitor and evaluate the progression of anterior segment diseases. By analyzing images of the anterior segment taken at different times, the model can identify subtle changes in lesions and provide clinicians with information on the progression of the disease. Deep learning has also shown great performance in the diagnosis of retinal diseases, being able to identify a variety of lesions such as diabetic retinopathy, glaucoma, and age-related macular degeneration. For example, convolutional neural networks (CNN) can analyze color fundus photographs to automatically detect lesion areas. Deep learning models can also accurately segment lesion areas in retinal images, such as retinal vessel segmentation and optic disk segmentation, which helps doctors more accurately assess the extent and severity of the lesions. Retinal imaging equipment is showing a trend toward “integration” and “low cost.” Multimodal deep learning technology, by integrating data from multiple imaging modalities (such as color fundus photographs, OCT, fluorescein angiography, etc.), can more comprehensively evaluate retinal lesions. But deep Learning has still some limitations. DL models require large, diverse datasets to generalize effectively. Bias in training data (e.g., underrepresented ethnic groups) may compromise diagnostic accuracy. Some models hinder clinical trust. For example, Eye-AD achieves AUC = 0.93 for Alzheimer’s detection but lacks transparency in feature selection. Complex architectures demand high computational power, limiting accessibility in low-resource settings.

Fundus aging is a complex biological process that involves degenerative changes in various tissues such as retinal blood vessels, nerve cells, and retinal pigment epithelium. Traditional single-modal analysis methods such as retinal image analysis alone) can provide some diagnostic information, but they have limitations in predicting the early stages of fundus aging and potential disease risk. For example, analysis based solely on fundus photography is difficult to capture changes in the microstructure of the retina and the characteristics of aging at the molecular level. Multimodal integration (e.g., OCT, fundus vascular imaging, and proteomic data combined) can significantly improve predictive performance, such as the VisionFM model, which improves the accuracy of age prediction by 20% by fusing multimodal imaging data and can identify molecular risk markers for diseases such as AMD at an early stage. Multimodal analysis can more comprehensively reflect the physiological and pathological state of the fundus by integrating multiple data sources. For example, combining fundus images with proteomic data from liquid biopsies allows for simultaneous assessment of structural changes in the retina and markers of aging at the molecular level. This method not only improves the accuracy of prediction of fundus aging but also enables early detection of potential disease risks. In addition, multimodal analysis has been applied to predict the molecular age of the fundus. Using AI machine learning models, researchers have developed tools that can predict the molecular age of the eye based on proteomic data from eye fluids.

In the future, deep learning will more deeply integrate medical imaging data from multiple modalities, further improving the accuracy and comprehensiveness of diagnosis. For example, combining various imaging techniques for the anterior segment of the eye and the retina will provide more precise diagnosis for complex ophthalmic diseases. Currently, the “black box” nature of deep learning models is a major challenge in their clinical application. Researchers will strive to enhance the interpretability of these models, enabling clinicians to better understand and trust the diagnostic results produced by them. Deep learning holds promise for advancing personalized ophthalmic care. By analyzing large amounts of patient data, models can predict disease progression and treatment responses in individual patients, thereby providing personalized treatment plans. The interdisciplinary collaboration between ophthalmology and fields such as computer science, physics, and mathematics will continue to deepen. Interdisciplinary teams will jointly develop more efficient and accurate deep learning models, driving continuous innovation in ophthalmic artificial intelligence technology.

### 7.1 Ethical considerations and data privacy in AI-driven diagnostics

The integration of AI-driven diagnostics in ocular aging research raises important ethical considerations, particularly concerning data privacy and security. The use of deep learning models often requires large datasets of patient images and biomarker data, which may include sensitive health information. Ensuring the anonymization and secure storage of such data is paramount to protect patient confidentiality. Additionally, the potential for algorithmic bias must be addressed to ensure equitable diagnostic outcomes across diverse populations. Transparent reporting of model performance and validation in independent cohorts can help mitigate these concerns and foster trust in AI-driven diagnostic tools.

## 8 Conclusions and perspectives

Advances in deep learning imaging technology have shown that retinal photograph-based deep learning (DL) algorithms are able to predict biological age and stratify the risk of death and major diseases in the general population. This technique offers a new, alternative way to measure aging. Deep learning models using ophthalmic images, such as OCTA, have shown potential for accurate identification and rapid screening in large-scale populations in the detection of early neurological cognitive dysfunction diseases. The combination of liquid biopsy proteomics and artificial intelligence enables the identification of cellular drivers of ocular aging and disease *in vivo*, and machine learning is increasingly being used in the field of liquid biopsy research through the integration of multi-omics data for multiplexing of ocular aging biomarkers to build models that outperform traditional and existing diagnostic methods. With the development of deep learning technology, the role of non-invasive diagnostic methods such as retinal imaging and liquid biopsy in early disease detection will become increasingly important, especially in resource-limited settings. Future studies can further explore and validate the biomarkers found in liquid biopsies, as well as how they behave and change in different disease states.

### 8.1 Summary of key applications of deep learning in ocular aging

• **Anterior Segment Aging:** Deep learning models have been successfully applied to automate the diagnosis of cataracts, presbyopia, and pterygium, improving accuracy and efficiency in clinical settings. These models leverage anterior segment imaging to detect subtle structural changes associated with aging.
• Fundus Aging: Multimodal deep learning approaches combining OCT, fundus photography, and OCTA have enhanced the early detection of retinal aging and neurodegenerative diseases. These tools provide a comprehensive assessment of retinal health, enabling timely interventions.

### 8.2 Future directions

• **Biomarker Validation:** Further research is needed to validate the specificity and sensitivity of newly identified biomarkers in diverse populations.
• **Integration of Multi-Omics Data**: Combining genomics, proteomics, and metabolomics with imaging data could provide a more holistic understanding of ocular aging.
• **Ethical AI Development:** Continued efforts to address data privacy and algorithmic bias will be critical for the widespread adoption of AI-driven diagnostics.
